# A comprehensive prognostic and immunological analysis of telomere-related lncRNAs in kidney renal clear cell carcinoma

**DOI:** 10.18632/aging.205056

**Published:** 2023-10-16

**Authors:** Ji Chen, Dong Zhang, Xiangbin Ren, Peng Wang

**Affiliations:** 1 Department of Urology, Shandong Provincial Hospital Affiliated to Shandong First Medical University, Jinan, China

**Keywords:** kidney renal clear cell carcinoma, telomere, long noncoding RNA, prognostic signature, immune environment

## Abstract

Kidney renal clear cell carcinoma (KIRC) is one of the most prevalent malignant tumors of the urinary system, with a high recurrence and metastasis rate. Telomeres and long non-coding RNAs (lncRNAs) have been documented playing critical roles in cancer progression. However, the prognostic significance of telomere-related lncRNA (TRLs) in KIRC is less well-defined. The Cancer Genome Atlas database was applied to retrieve the expression profiles and corresponding clinical information of KIRC patients. To create the TRLs prognostic signature, univariate Cox regression, least absolute shrinkage and selection operator analyses were performed. The prognostic signature, comprised of nine prognostic TRLs, was developed and demonstrated superior prognostic ability for KIRC patients. Additionally, the risk score acted as an independent prognostic indicator. A nomogram incorporating age, grade, stage, and signature-based risk scores was also developed and exhibited excellent predictive accuracy. Several immune activities were associated with the signature, as determined by gene function analysis. Further analysis revealed differences in the status of immunity and the tumor microenvironment between low- and high-risk groups. Notably, KIRC patients with high-risk scores were more responsive to immunotherapy and chemotherapy. To summarize, our study developed a new prognostic signature consisting of nine telomere-related lncRNA that can precisely predict the prognosis of KIRC patients. The signature was shown to be of substantial value for the tumor microenvironment and tumor mutation burden, thereby contributing to a framework for the individualized treatment of patients.

## INTRODUCTION

Renal cell carcinoma (RCC), one of the most prevalent tumors in urology, accounts for about 3% of all adult malignancies and over 90% of all renal malignancies [1]. Kidney renal clear cell carcinoma (KIRC) is the most prevalent subtype with the highest mortality rate and accounts for approximately 70% of all kidney cancers [2]. Estimated new cases and deaths in the United States in 2022 were 97000 and 13980, respectively [3]. Nearly one-third of KIRC patients have metastases by the time of diagnosis [4]; this is due to the lack of early symptoms and reliable screening markers. Surgical resection is the standard treatment for early-stage disease; however, 30% of patients will experience recurrence or metastasis after surgery [5]. A substantial proportion of patients with advanced KIRC exhibited poor outcomes, with a 5-year survival rate is only 10% [4]. Despite advances in clinical diagnostic technology and molecular targeted therapy, including immune-checkpoint inhibitors and antiangiogenic agents, which increase the survival rate of advanced-stage patients, more than 100,000 patients still die each year [6–8]. Consequently, it is urgent to identify novel biomarkers to predict clinical outcomes and aid in decision-making.

Telomeres are nucleoprotein structures at the distal end of chromosomes, composed of a repeating TTAGGG sequence and a bound shelterin protein complex [9]. Telomeres are essential in maintaining the stability of chromosome structure and the genome [10]. Although telomeres shorten with each cell division, it has been reported that telomere abnormalities have been identified in certain disease states, such as dyskeratosis congenita, aplastic anemia [11], diabetes mellitus [12], and psychiatric disorders [13]. Moreover, many previous studies have reported the crucial role of telomeres in tumor initiation and progression [11, 14]. However, shortening telomeres can also serve dual functions in the process of cancer development. On the one hand, telomere shortening can result in genome instability and therefore promote the progression of cancer. Alternatively, telomere shortening has a tumor-suppressing effect by inhibiting cell proliferation [11, 15]. Regarding KIRC, several studies suggested that telomere length could predict kidney cancer survival, but its prognostic role is still unconfirmed [16].

Long non-coding RNAs (lncRNAs) are non-coding RNAs exceeding 200 nucleotides in length [17]. Several publications have reported that lncRNAs serve as an important marker for various human cancers [18], and are among the most sensitive and specific biomarkers for cancer. As a result, it is confirmed that lncRNA can be utilized to predict clinical prognosis and direct treatment in KIRC [19]. Nevertheless, the functions of telomere-related lncRNAs (TRLs) in KIRC require further investigation. Given the significance of telomeres and lncRNAs, the use of novel methods to provide an improved prognosis for KIRC patients may offer a viable strategy.

The previous investigations have chiefly focused on the telomere length in cancers and their role in cancer prognosis. Now, there is still no study to investigate the prognostic values of TRLs in cancers so far. Herein, we constructed a prognostic signature based on TRLs to predict the outcomes of KIRC and then explored the potential role in the selection of therapeutic agents.

## RESULTS

### The expression of telomere-related genes and identification of TRLs

**Figure 1:**
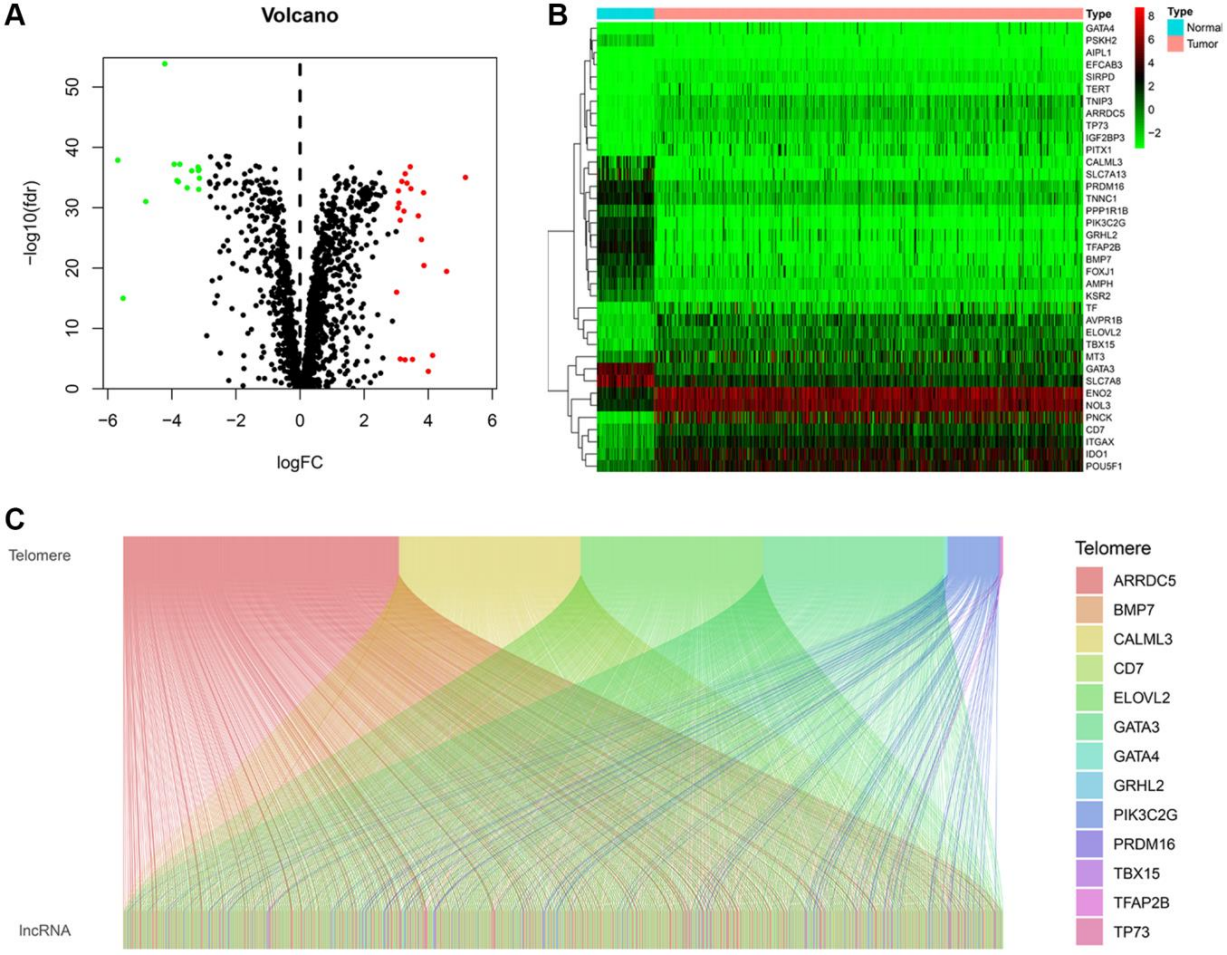
**Identification of telomere-related lncRNAs (TRLs) in KIRC.** (**A**) Violin plot showing the differences in the expression of telomere-related genes between normal and KIRC tissues. (**B**) Heatmap showing the expression profile of differentially expressed telomere-related genes. (**C**) Sankey graph of the co-expression networks of the TRLs and telomere-related genes.

The study flow diagram is shown in (Supplementary Figure 1). We obtained the expression profiles of 2088 telomere-related genes telomere from 72 normal kidneys and 539 KIRC tissues. In KIRC, 37 telomere-related genes exhibited significant expression differences, including 15 down-regulated and 22 up-regulated genes (Figure 1A, 1B). The expression profiles of 37 differentially expressed telomere-related genes and lncRNAs were then utilized to conduct a Pearson correlation analysis. Under the conditions of |R| > 0.7 and *P* < 0.001, 13 telomere-related genes were retained and 648 lncRNAs were identified as TRLs. The co-expression networks between telomere-related genes and TRLs were illustrated in the Sankey diagram (Figure 1C).

### Construction of TRLs prognostic signature

**Figure 2:**
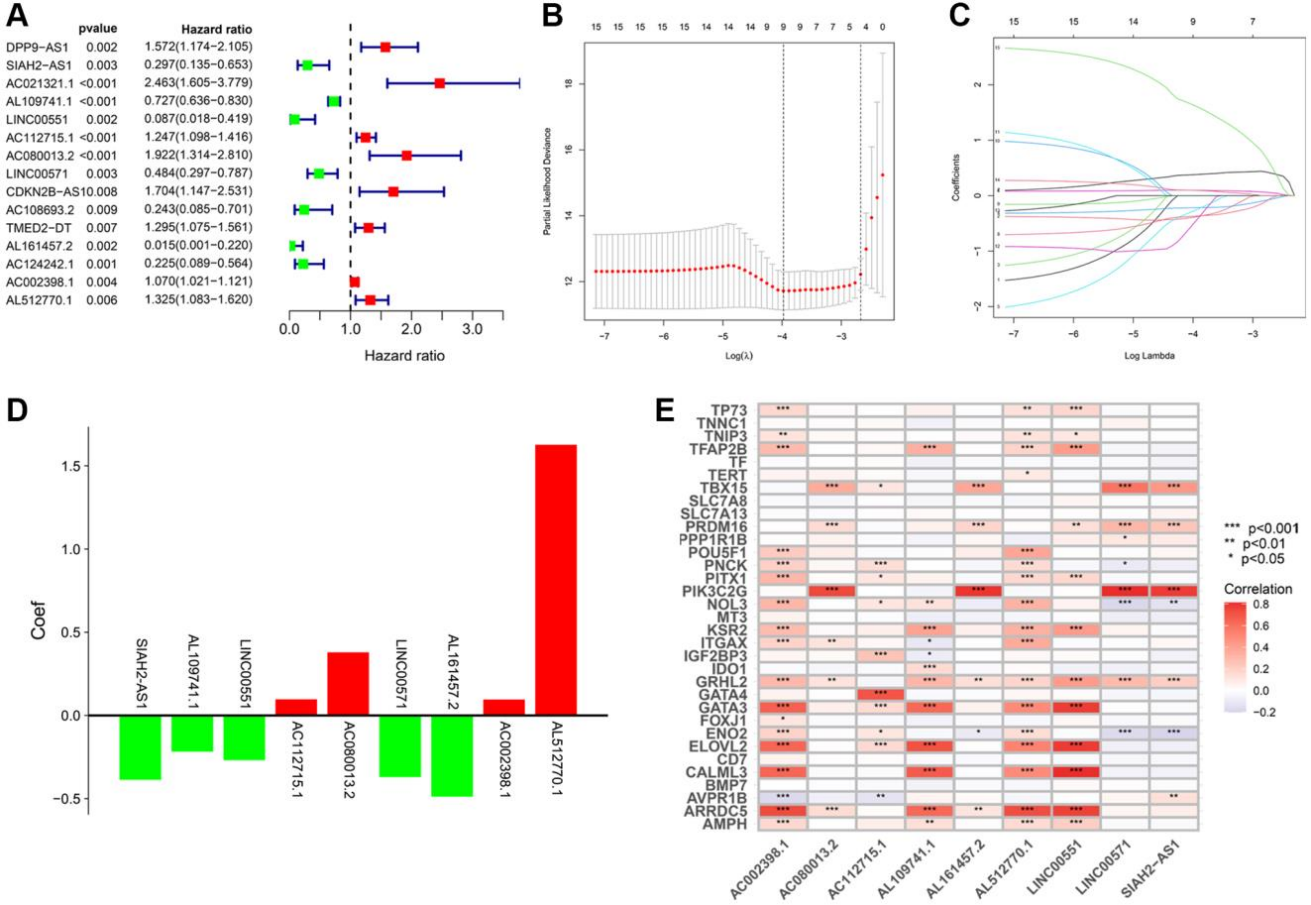
**Construction of TRLs prognostic signature in KIRC.** (**A**) The forest plot shows 15 prognostic TRLs using univariate Cox regression analysis. (**B**) The 10-fold cross-validation for the selection of optimal parameter lambda in the LASSO regression. (**C**) The profile of the LASSO coefficient. (**D**) The coefficient distributions of nine TRLs. (**E**) Heatmap for co-expression of signature-based TRLs and telomere-related genes.

The univariate Cox analysis revealed that 15 of 648 TLRs were significantly related to overall survival (OS) (Figure 2A). In the training cohort, the aforementioned genes were subjected to the least absolute shrinkage and selection operator (LASSO) regression analysis for dimensionality reduction. There was a total of nine TRLs that were filtered out and used to create a prognostic signature (Figure 2B, 2C). The coefficient distributions of nine TRLs are illustrated in Figure 2D. The risk score for each KIRC patient can be estimated: risk score = (−0.387 × value of SIAH2-AS1) + (−0.218 × value of AL109741.1) + (−0.269 × value of LINC00551) + (0.097 × value of AC112715.1) + (0.379 × value of AC080013.2) + (−0.370 × value of LINC00571) + (−0.488 × value of AL161457.2) + (0.096 × value of AC002398.1) + (1.627 × value of AL512770.1). Additionally, we conducted a co-expression analysis of TRLs and telomere-related genes, and the results indicated that AC002398.1 and AL512770.1 co-expressed more than any other TRLs and telomere-related genes, which were linked to a poor prognosis (Figure 2E).

### Validation of TRLs prognostic signature

**Figure 3:**
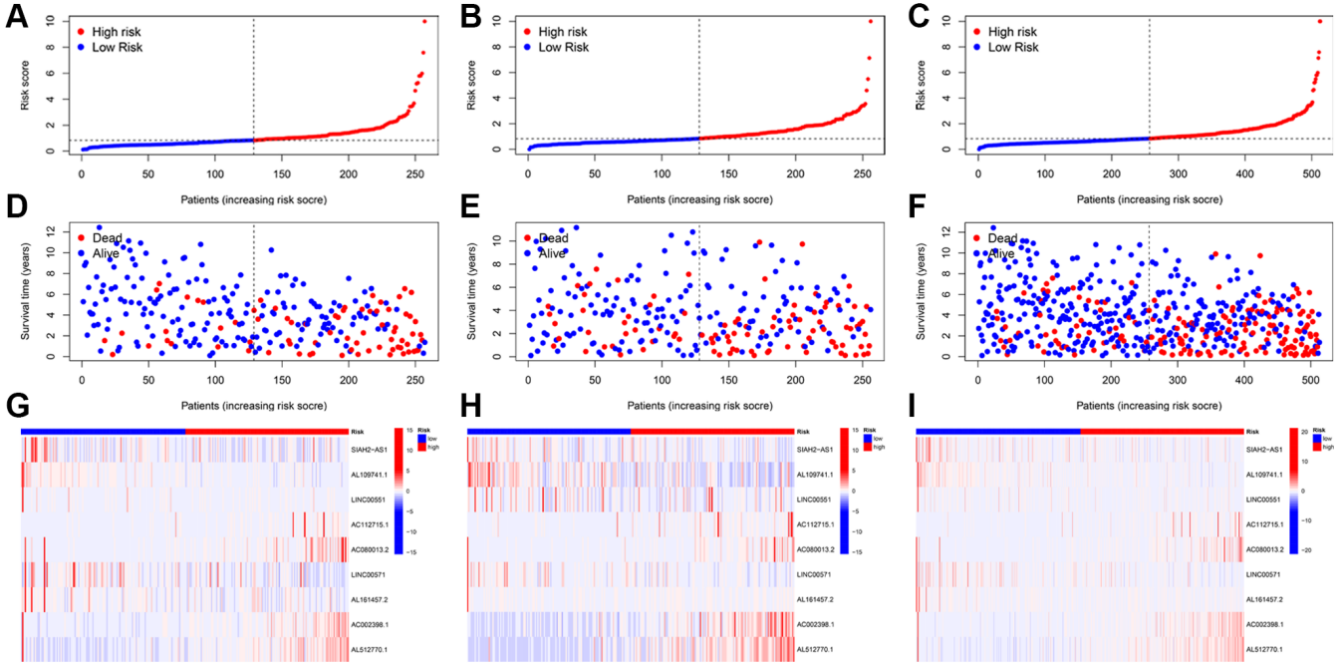
**Prognosis value of the TRLs prognostic signature in the training; testing; and entire cohorts; respectively.** (**A**–**C**) The distributions of risk scores. (**D**–**F**) The survival time and survival status. (**G**–**I**) Heatmaps of 9 TRLs expression.

KIRC patients were separated into low- and high-risk groups using the median score. Subsequently, we ranked the KIRC patients based on their risk scores from the training, testing, and entire cohorts (Figure 3A–3C). We then compared the patient survival status and the expressions of nine TRLs between two groups in the training, testing, and entire groups, respectively. We found that as the risk score increases, the number of deaths among patients with KIRC increased (Figure 3D–3F). Furthermore, four deleterious lncRNAs (AC112715.1, AC080013.2, AC002398.1, and AL512770.1) exhibited relatively high expression in the high-risk group, whereas five protective lncRNAs (SIAH2-AS1, AL109741.1, LINC00551, LINC00571, and AL161457.2) demonstrated the opposite trend (Figure 3G–3I).

**Figure 4:**
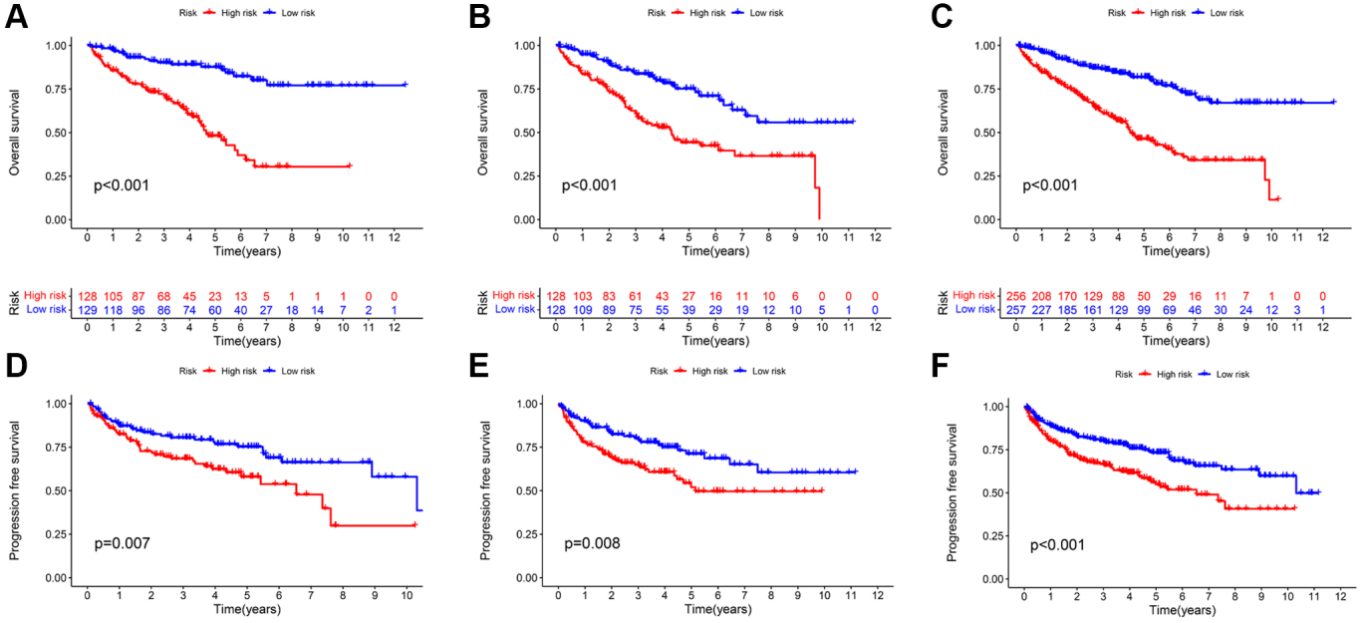
**Kaplan-Meier survival analysis based on TRLs prognostic signature.** The OS rates (**A**–**C**) and PFS rates (**D**–**F**) of different risk groups in the training; testing; and entire cohorts; respectively.

The survival analysis revealed that the high-risk group suffered a significantly shorter OS rate than the low-risk group (Figure 4A–4C). In addition, there was a significant difference in PFS between the two groups, with the progression-free survival (PFS) rate being higher in the low-risk group (Figure 4D–4F). Except for stage N1, significant statistical differences in survival analysis were observed between KIRC patients with distinct clinical features (Supplementary Figure 2). The absence of a significant difference in the OS rate may be partially explained by the fact that there are fewer cases of N1 group. In addition, we found that patients with high-grade and advanced-stage tumors had significantly higher risk scorers than those with low-grade and early stages tumors (Supplementary Figure 3). Between these two groups, there was no significant difference in the distribution of age and gender. The preceding result revealed that the constructed prognostic signature holds for almost all clinical features of KIRC patients.

**Figure 5:**
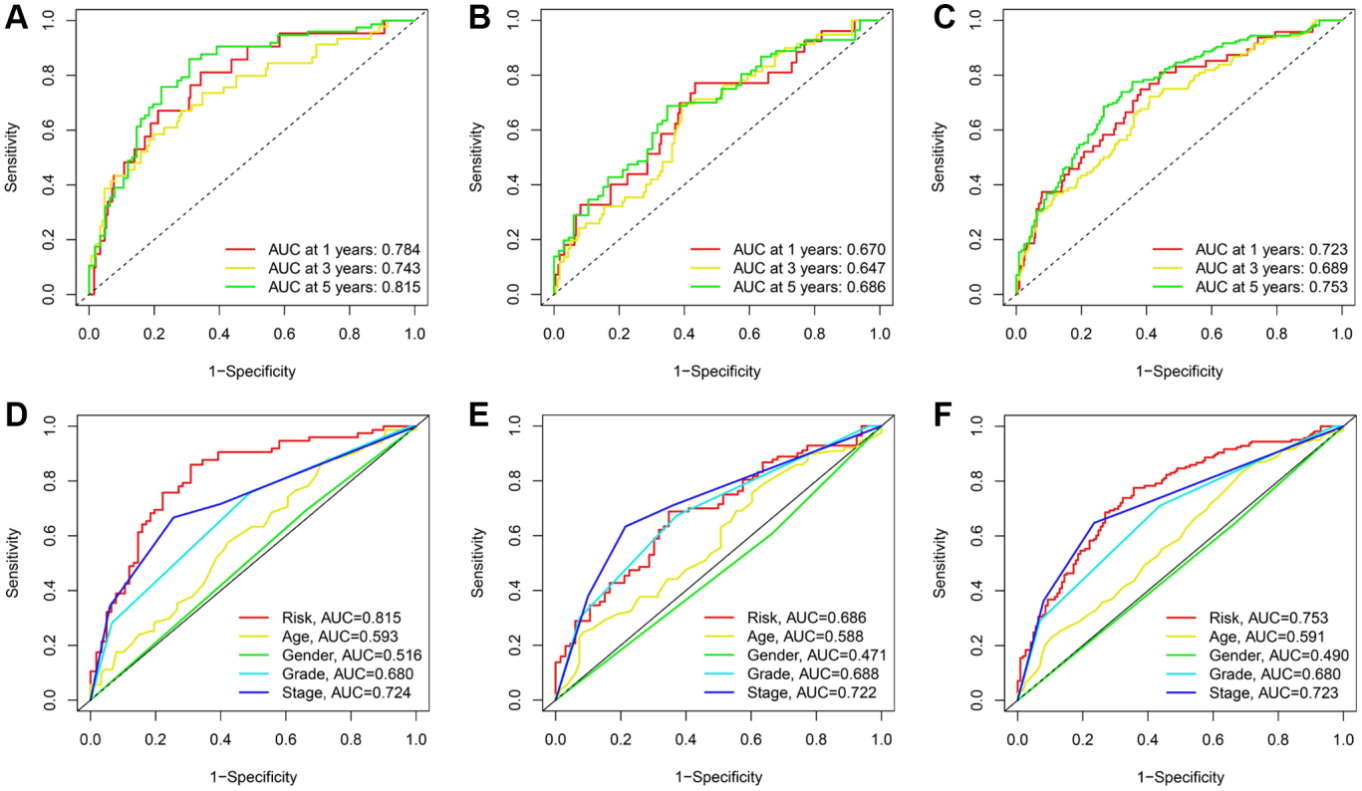
**ROC curves for TRLs prognostic signature in the training; testing; and entire cohorts; respectively.** (**A**–**C**) Time-dependent ROC curves for the prognostic signature. (**D**–**F**) Multi-parametric ROC curve analysis for the signature-based risk score and clinical features.

The predictive efficacy of the signature was further evaluated using the area under the curve (AUC) and its values for 1, 3, and 5 years were 0.673, 0.743, and 0.893, respectively (Figure 5A). Comparatively, the maximum AUCs for the testing cohort and the entire cohort were 0.686 and 0.753, respectively (Figure 5B, 5C). In addition, we observed that the risk score predicted the prognosis of KIRC patients better than other clinical variables (Figure 5D–5F).

Principal component analyses (PCA) were utilized to examine the distinct distribution patterns between two groups using a variety of data sets, including whole genome data, differentially expressed telomere-related genes, TRLs, and signature-based TRLs (Supplementary Figure 4). Using the expression of signature-based TRLs, the two groups tended to separate in two directions, whereas the other three expression profiles failed to distinguish between the groups. These results demonstrated that this signature possessed strong predictive ability.

### The independence of TRLs prognostic signature

**Figure 6:**
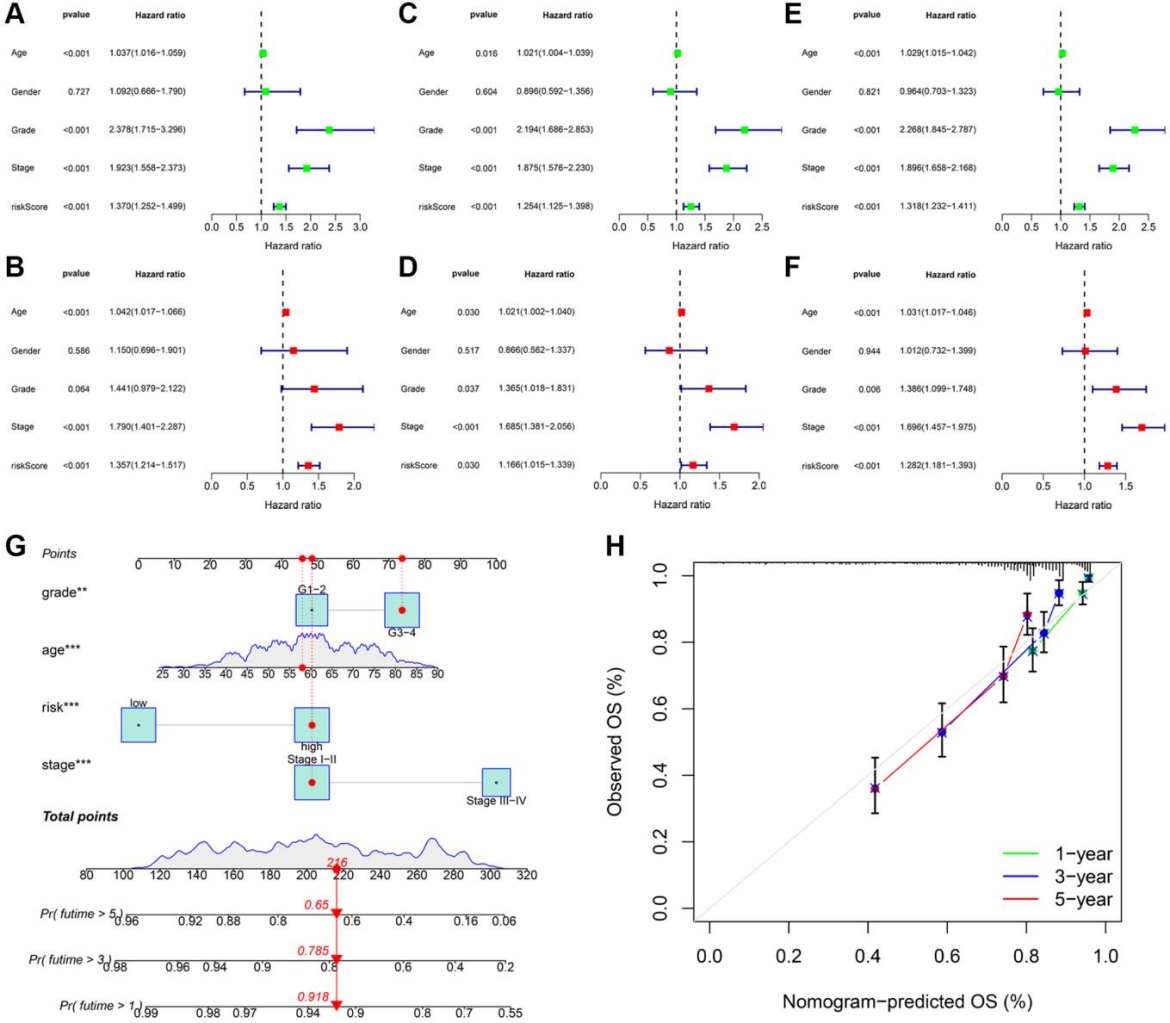
**Independent prognostic validation of risk signatures and development of clinical nomogram.** Univariate and multivariate Cox regression analysis results were presented in the form of forest plots in the training (**A**, **B**); testing (**C**, **D**); and entire (**E**, **F**) cohorts; respectively. (**G**) Nomogram to quantitatively predict 1; 3; and 5-year OS in KIRC patients. (**H**) The calibration curves of this nomogram.

We utilized univariate and multivariate Cox analyses of variables to determine if the signature-based risk score was an independent prognostic factor for OS, independent of other clinical characteristics. In the training cohort, univariate Cox analysis revealed a significant association between risk score and patient survival (Figure 6A). In addition, a multivariate Cox analysis implied that the risk score was an independent prognostic factor (Figure 6B). The results of independent prognostic analysis for the testing (Figure 6C, 6D) the entire cohorts (Figure 6E, 6F) were identical. Accordingly, these findings suggested that the signature-based risk score was an independent prognostic factor for OS, suggesting which had a certain clinical utility for the prognosis evaluation. Subsequently, we developed a nomogram by integrating three independent indicators (age, grade, and stage) and a risk score to predict the 1, 3, and 5-years OS of KIRC patients (Figure 6G). The calibration curves validated the consistency of the nomogram (Figure 6H).

### Functional enrichment analysis

Considering that the outcomes of KIRC patients in low- and high-risk groups differed significantly, GO (Gene Ontology) and KEGG (Kyoto Encyclopedia of Genes and Genomes) analyses were conducted to investigate the potential biological function of 534 genes with differential expression between the two groups. GO annotation revealed that these genes were significantly associated with biological behaviors like humoral immune response (Supplementary Figure 5A–5C). KEGG revealed that these genes were related to pathways including glycerophospholipid metabolism. (Supplementary Figure 5D, 5E). These biological behaviors and pathways are probably responsible for the high-risk group’s tendency to have poorer clinical outcomes.

### Immune landscape of KIRC patients

**Figure 7:**
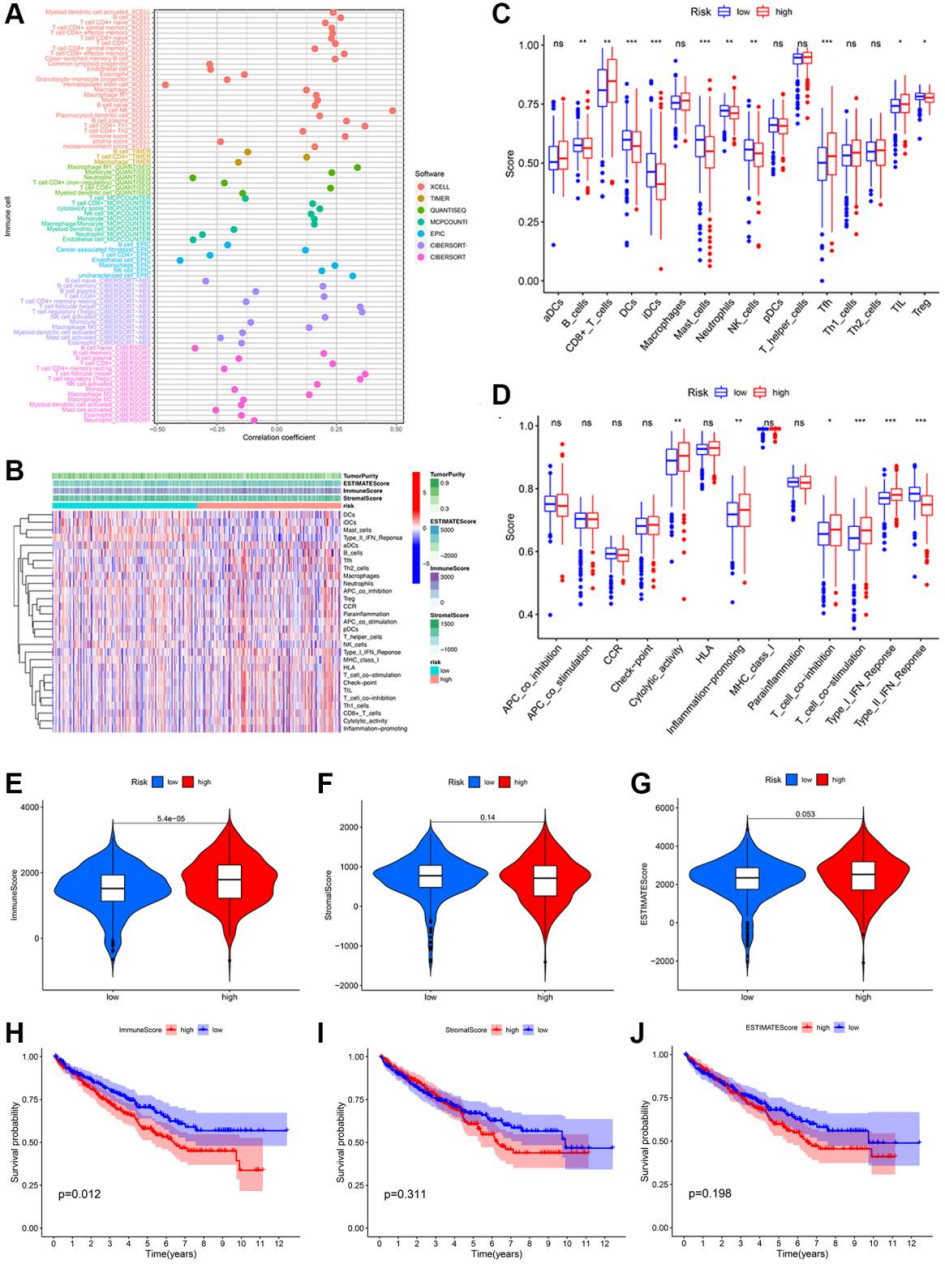
**Potential effects of prognostic signatures on tumor immune microenvironment in KIRC patients.** (**A**) The correlation between different immune cells and risk scores was analyzed using seven algorithms. (**B**) The heatmap of immune cells and immune-related pathways. Differences in enrichment scores of 16 immune cells (**C**) and 13 immune-related pathways (**D**) between the low- and high-risk groups. Distribution (**E**–**G**) and Kaplan-Meier survival (**H**–**J**) of immune score; stromal score; and ESTIMATE score between the low- and high-risk groups.

Since the preceding results indicated a close association between risk scores and immune functions, we investigated the immune profiles of KIRC patients in low- and high-risk groups. A significant correlation between various immunocytes and the risk score was observed, which was determined by employing seven algorithms to study the relationship between risk score and tumor immune cell infiltration (Figure 7A). Then, using the single sample Gene Set Enrichment Analysis (ssGSEA) algorithm, we quantified scores for immune cell enrichment and function enrichment (Figure 7B). The proportions of CD8+ T cells, T follicular helper cell and tumor Infiltrating lymphocytes were higher in the high-risk group than the low-risk group (Figure 7C). As for the immunization-associated function enrichment scores, cytolytic activity, inflammation promoting, T cell co-inhibition/stimulation, and IFN response (Type 1) were higher in the high-risk group than the low-risk group (Figure 7D). We then determined that the immune score of the high-risk group was obviously higher than the low-risk group using the ESTIMATE algorithm (Figure 7E). Neither the stromal score nor the estimated score varied significantly between subgroups (Figure 7F, 7G). In addition, the poor prognosis was more prevalent among KIRC patients with higher immune scores (Figure 7H), indicating that the immune score can be used to predict the outcomes of these patients. However, there was no significant survival difference in the stromal score and estimate score (Figure 7I, 7J).

### Tumor mutation analysis

**Figure 8:**
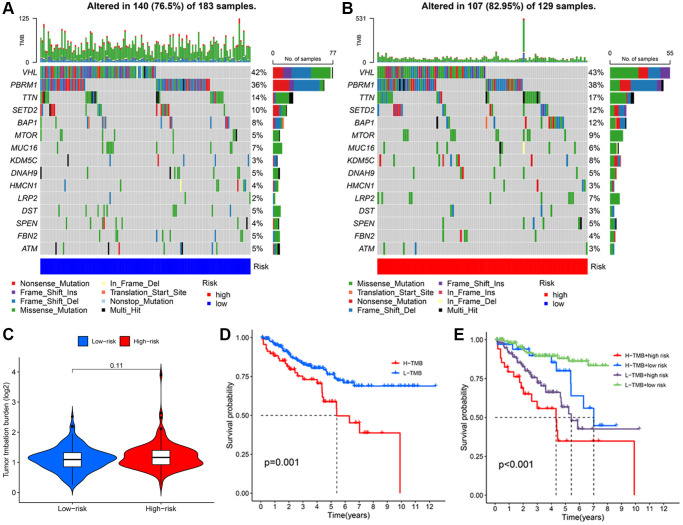
**Analysis of TMB based on TRLs prognostic signature in KIRC patients.** The waterfall plot illustrates the type and frequency of tumor mutational burden in the low-risk group (**A**) and high-risk group (**B**). (**C**) TMB levels between two groups. (**D**) The Kaplan-Meier curve of KIRC patients in the low- and high-TMB groups. (**E**) The Kaplan-Meier curves of KIRC patients among diverse subgroups based on the risk score and TMB levels.

To characterize tumor mutation analysis (TMB) in KIRC patients, we determined the TMB value for each individual case. The probability of a genetic mutation occurring in the low-risk group and high-risk group were 76.50% and 82.95%, respectively (Figure 8A, 8B). However, there is no significant difference in the value of TMB (Figure 8C). To investigate the value of TMB in KIRC, we then conducted survival analyses for different TMB groups and risk groups. The patient with a low TMB represented favorable OS than the patient with a high TMB (Figure 8D). The synergistic effect of TMB concentrations and risk scores on outcome prediction were then investigated in greater detail. Combining TMB subgroup analysis with the risk score revealed statistically significant differences in survivability across groups (Figure 8E). Additionally, the low-TMB combining low-risk group had the highest OS rate, whereas the high-TMB combining high-risk group had the lowest OS rate. These findings indicated that the signature-based risk score combined with TMB had a good prognostic value. Comparing the high-risk group to the low-risk group, the high-risk group exhibited a relatively higher immune status.

### Immunotherapy responsiveness and drug sensitivity analysis

**Figure 9:**
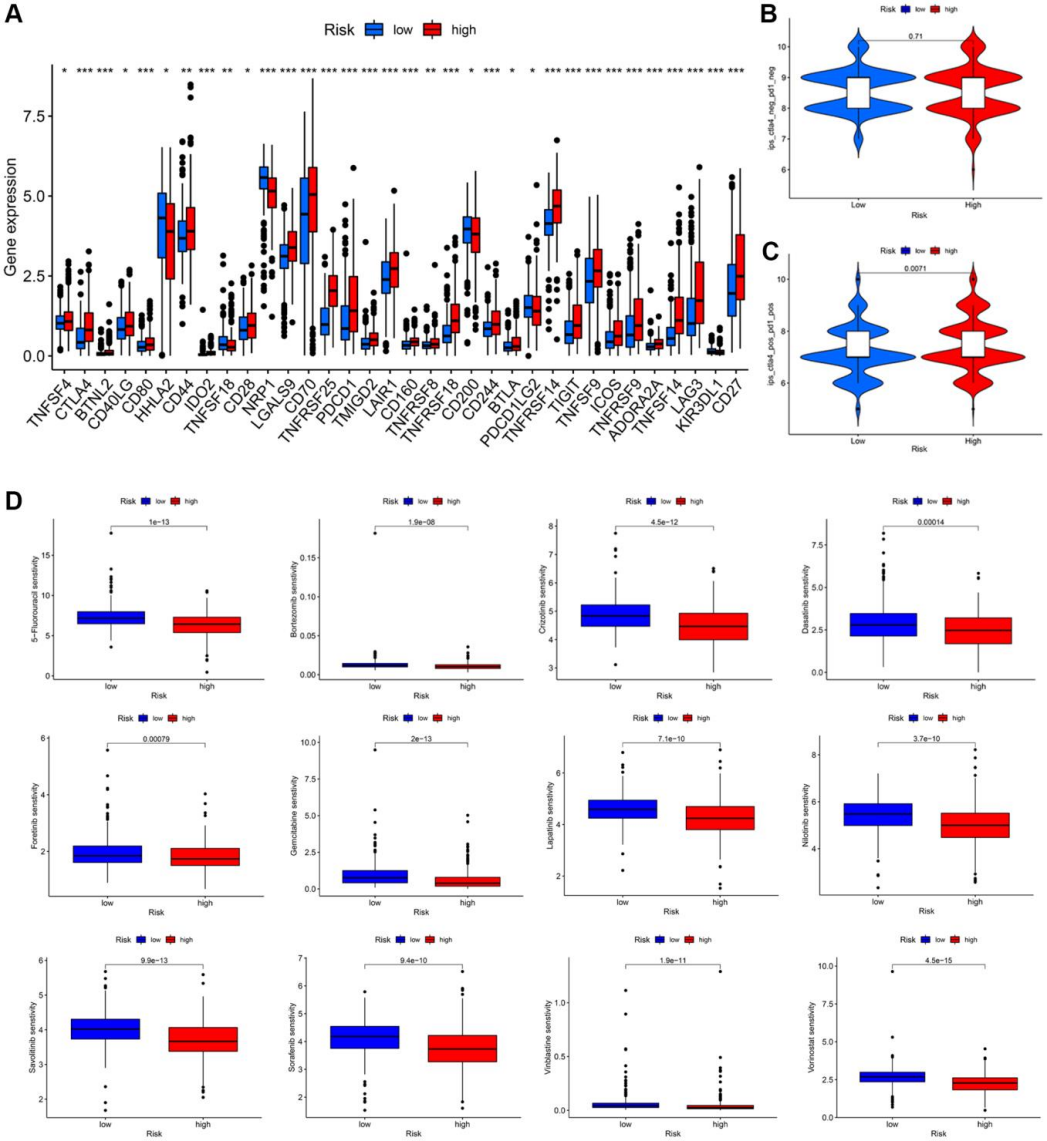
**Immunotherapeutic response prediction and drug susceptibility analysis between low- and high-risk groups in KIRC patients.** (**A**) Differential expression of common immune checkpoints. (**B**, **C**) Relative response of anti-PD1 and anti-CTLA4 therapy. (**D**) Difference analysis of anti-tumor drug sensitivity.

Subsequently, we determined the immune checkpoint inhibitors (ICIs) expression levels and drug sensitivity of KIRC patients. In the high-risk group, a number of ICIs, including PD-1, were upregulated (Figure 9A). Examining the differences in immunotherapy efficacy between the two groups, we then obtained the IPS of the KIRC patients from the Cancer Immunome Atlas (TCIA) database. Overall, there was no statistical difference in the IPS of CTLA4_neg_PD1_neg_ (Figure 9B); however, there existed difference in the group of CTLA4_pos_PD1_pos_ (Figure 9C), indicating that immunotherapy may be more suitable for the high-risk group. By comparing differences in the maximum 50% inhibition concentration (IC50) values of several drugs between two groups, we discovered that our signature may influence drug sensitivity to some extent. The fact that the IC50 values of 12 drugs in the low-risk groups were higher than those in the high-risk groups suggests that KIRC patients with high-risk scores may be more sensitive to the aforementioned chemical therapy (Figure 9D). The findings demonstrated that this prognostic signature has a certain guiding significance for selecting specific therapy for KIRC patients.

## DISCUSSION

KIRC is one of the most prevalent tumors, particularly in patients with advanced disease, whose prognosis is often very unsatisfactory [5, 6, 20]. Research has shown that lncRNAs perform crucial roles in tumor progression. In addition, aberrant lncRNA expression has been detected in KIRC [21, 22]. There are reports that prognostic models based on LncRNA can accurately predict the prognosis of KIRC patients. The research conducted by Zhou resulted in the development of a risk assessment model comprised of pyroptosis-related lncRNAs that could predict the outcomes of KIRC patients [23]. Xu et al., constructed a prognosis signature using 10 cuproptosis–related lncRNAs, which can assist in determining the prognosis and molecular profile of KIRC patients [24]. Zhang et al., found that the epithelial-mesenchymal transition-related lncRNA prognostic signature is significant for KIRC prognosis [25]. Telomeres are essential for controlling genome stability because they maintain the integrity of chromosomes [14]. Interestingly, several types of cancer have been linked to aberrant telomeric structures, indicating their significance in the development and progression of cancer. However, the purpose of TRLs in KIRC was poorly understood.

In the present study, 37 telomere-related genes with differential expression between normal and KIRC tissues were initially screened. 15 of the 648 TRLs identified by Spearman correlation analysis were considered prognostic lncRNAs in univariate Cox analysis. Based on LASSO regression analysis, a prognostic signature consisting of nine TRLs was constructed. Among them, SIAH2-AS1 was found to be expressed at low levels in breast cell lines relative to normal cells and was associated with a favorable prognosis [14]. Wang et al., identified LINC00551 as a tumor suppressor in lung adenocarcinoma and demonstrated that LINC00551 inhibits glycolysis via regulating c-Myc-mediated PKM2 expression [26]. However, a recent study presented contradictory results. Through the regulation of the miR-98-5p/TGFBR1 axis, Li et al., found that LINC00551 may promote postoperative distant recurrence in non-small-cell lung cancer [27]. LINC00571 was identified as a risk factor with HR >1 for patients with triple-negative breast cancer, and high LINC00571 expression was associated with poor overall survival [28]. Regarding the remaining six TRLs, however, there were few reported cases in the literature.

We did not only obtained the most accurate signature, but we also calculated the optimal cutoff value for separating patients with KIRC into low- or high-risk groups. Analysis of survival revealed that the clinical outcomes of KIRC patients in the high-risk group were poorer relative to those of patients in the low-risk group. Moreover, the prognostic signature was applied to nearly all clinical features of KIRC patients in subgroup analyses. The ROC analysis revealed that the signature is superior to other clinical characteristics for predicting prognosis. The univariate and multivariate cox regression analyses further determined the signature's independent prognostic function. Moreover, the independent clinical indicators were integrated to develop a nomogram, thereby making the signature more applicable to clinical practice.

GO annotation and KEGG analysis were performed to increase understanding of the potential biological behaviors and mechanisms of the involved signature. The results showed that many enrichment biological functions were significantly associated with immune activities. In addition, several biological phenomena, including humoral immune response, immunoglobulin production, and immunoglobulin complex, may be involved in the process. Glycerophospholipid metabolism [29] and the NF-kappa B signaling pathway [30] may partially explain why KIRC patients with high-risk scores had a worse prognosis.

Taking into account the results of function enrichment and the fact that KIRC was an immunogenic tumor, we investigated the associations between signature and the immune microenvironment of KIRC tumors and noticed that multiple immune cells differed between the different groups. Importantly, we found that the high-risk group had a higher proportion of CD8+ T cells, which was associated with a poor prognosis due to immune evasion via T cell exhaustion [31, 32]. Besides, most of the immunization-associated function enrichment scores, including cytolytic activity, inflammation-promoting, T cell co-stimulation/inhibition, and INF response, were higher in the high-risk group. In addition, we observed that the immune score was higher in the high-risk group compared with the low-risk group. In addition, KIRC patients at high risk with elevated immune scores had a dismal survival rate, which was consistent with previous research [33]. The aforementioned findings indicated that the constructed prognostic signature might reflect the immune status of the tumor microenvironment in patients with KIRC.

TMB levels are increasingly regarded as a sensitive predictor of the clinical response to immunotherapy in multiple cancers [34]. We analyzed somatic mutations to explore the role of TMB in KIRC. A total of 247 patients possessed somatic mutations, 107 (76.50%) in the low-risk group and 140 (85.95%) in the high-risk group. Similarly, to other studies, the VHL genes exhibited the highest frequency of mutations. In addition, we discovered that the high TMB group suffered poor survival rates, although there was no difference between TMB and risk score. This phenomenon was also identified in a separate study [35].

KIRC is currently one of the most prevalent cancer immunotherapies used to treat advanced-stage cancer. Although PD1 (PDL1) can prolong the survival in patients with metastatic KIRC, only a minority of patients benefit from them [36]. Accordingly, we hope that our signature will provide guidance for individualized immunotherapy. Then, we analyzed the expression of ICIs and found that the majority of ICIs were higher in the high-risk group. These findings suggested that KIRC cases with high-risk scores may be more sensitive to the treatment of ICIs, which may be advantageous for achieving greater therapeutic effects. Recent FDA approvals of α-PD-1 in combination with α-CTLA-4 are transforming the standard of care for metastatic KIRC [36, 37]. Using TCIA data to predict the efficacy of immunotherapy, we found that the scores of IPS−PD1−CTLA4 blocker were higher in the high-risk group, indicating that this signature may be a potential biomarker for evaluating the immunotherapy response in KIRC patients. In addition, we investigated the response of patients to chemotherapy sensitivity as measured by IC50 values and found that the low-risk group was more sensitive to several drugs. These findings may provide KIRC patients with a viable treatment option.

The study also had a number of limitations. First, the retrospective nature of our study necessitated a prospective multicenter investigation to validate the preliminary findings. Second, experimental studies of a fundamental nature are required to elucidate the mechanism by which TRLs influence the progression of KIRC. Third, some prognostic lncRNAs may have been overlooked because we only considered a single phenotype when constructing a prognostic signature. Despite a number of flaws, the clinical applicability of the present study is extremely promising.

To summarize, our study developed a new prognostic signature consisting of nine telomere-related lncRNA that can precisely predict the prognosis of KIRC patients. The signature was shown to be of substantial value for the tumor microenvironment, tumor mutation burden, and drug sensitivity, thereby contributing to a framework for the individualized treatment of patients.

## MATERIALS AND METHODS

### Data collection

The RNA sequencing data (FPKM value) of 539 KIRC and 72 normal patients were downloaded from The Cancer Genome Atlas (TCGA) database (https://portal.gdc.cancer.gov/). Individuals with incomplete RNA expression data, missing prognostic information, or a 30-day survival time were excluded from our study. The 513 KIRC cases were randomly separated into two groups (1:1 ratio) for training (*n* = 257) and testing (*n* = 256). Patient demographics and baseline characteristics were listed in Supplementary Table 1.

### Identification of telomere-related lncRNAs

TelNet is a user-friendly gene database designed to facilitate the study of genes involved in telomere maintenance mechanisms. Accordingly, the information on 2088 telomere-related genes were identified from TelNet (http://www.cancertelsys.org/telnet/) database [38]. With a preset threshold (|log2 fold change (FC)| ≥ 3.0 and false discovery rate (FDR) < 0.05), the “limma” package was used to identify the differentially expressed telomere-related genes [39]. The “limma” package was further used to conduct a Pearson correlation analysis on differentially expressed telomere-related genes and lncRNAs expression. When |correlation coefficient (R)| > 0.7 and *P* < 0.001, TRLs were eliminated from further consideration. A Sankey diagram was created to illustrate the relationship more intuitively between differentially expressed telomere-related genes and TRLs.

### Construction and validation of the TRLs-based prognostic signature

We merged TRLs expression data with corresponding survival data and then conducted a univariate Cox analysis to identify TRLs associated with OS (*P* < 0.05). In the training cohort, the LASSO regression analysis was used to develop the optimal prognostic signature. The risk score was estimated in the three cohorts using the following equation: $\text{Risk score}=\sum_{i=1}^{n}x(i)\times Coef(i).$ The *x*(*i*) and *Coef*(*i*) represented the expression values of TRLs and their coefficients, respectively. Using the median score, the patients were classified into low-risk (< median) and high-risk (≥median) groups. Additionally, Kaplan-Meier survival analysis was utilized to compare the OS and PFD between the two groups using log-rank tests. Moreover, the receiver operating characteristic (ROC) curves and subgroup analyses were conducted to further evaluate the signature’s ability to predict outcomes and the performance of relevant clinical parameters. To determine the independence of signature-based risk scores, Cox regression analyses were conducted. The aforementioned analyses were validated in the testing and entire cohorts. A nomogram was developed eventually by integrating all independent prognostic parameters to qualitatively predict the OS of KIRC patients across the entire cohort.

### Functional enrichment analysis

To investigate the potential molecular mechanisms underlying the prognostic signature, the differentially expressed genes between low- and high-risk groups were identified (|log2FC| > 1 and FDR < 0.05) using the “limma” package. The functional enrichment analysis of GO and KEGG was then carried out on the above genes. An adjusted *P* < 0.05 was regarded to be the cut-off point.

### Analysis of tumor immune microenvironment

Seven algorithms (CIBERSORT-ABS, CIBERSORT, EPIC, MCPCOUNTER, QUANTISEQ, TIMER, XCELL) were used for calculating the immune cell infiltration in various KIRC cases. The correlation between infiltration levels of immune cell and risk score was then determined using Spearman correlation analysis. In addition, the ssGSEA was employed for analyzing the relative abundance of immune populations between different groups [40]. Subsequently, we used the ESTIMATE algorithm to obtain the immune score for each KIRC patient and for comparing the score differences between risk groups [41].

### Tumor mutation analysis

Based on somatic mutation frequencies, TMB was calculated. The median TMB value was then used to divide the KIRC patients into low- and high-TMB groups. The waterfall plot of a mutational landscape was visualized through the “maftool” package [42]. In addition, comparative and survival analyses were conducted between two groups to investigate the difference in somatic mutation.

### Prediction of immunotherapy responsiveness and potential drug sensitivity

We also compared the expression levels of several ICIs genes between low- and high-risk groups and then utilized the TCIA database to predict potential immunotherapy responses [43]. Gene expression profiles and drug response data were obtained from the Genomics of Drug Sensitivity in Cancer (GDSC) database. Additionally, we adopted ridge regression to predict the IC50 of drugs with the “oncoPredict” package [44]. The IC50 was calculated as tolerance ability, and a higher IC50 indicated the cells showing higher resistance to drugs. The difference in sensitivity score was then analyzed between the two groups.

### Statistical analysis

All statistical analyses were performed in R statistical software (version 4.0.1) and Perl language (version 5.30.2). Unless otherwise specified, statistical significance was considered for two-tailed *P* < 0.05.

### Availability of data and materials

The datasets used and/or analyzed during the current study are available from the corresponding author on reasonable request.

## Supplementary Materials

Supplementary Figures

Supplementary Table 1

## References

[1] Meric-Bernstam F, Tannir NM, Iliopoulos O, Lee RJ, Telli ML, Fan AC, DeMichele A, Haas NB, Patel MR, Harding JJ, Voss MH, Owonikoko TK, Carthon B, et al. Telaglenastat Plus Cabozantinib or Everolimus for Advanced or Metastatic Renal Cell Carcinoma: An Open-Label Phase I Trial. Clin Cancer Res. 2022; 28:1540–8. 10.1158/1078-0432.CCR-21-2972 35140121 PMC9164172

[2] Harlander S, Schönenberger D, Toussaint NC, Prummer M, Catalano A, Brandt L, Moch H, Wild PJ, Frew IJ. Combined mutation in Vhl, Trp53 and Rb1 causes clear cell renal cell carcinoma in mice. Nat Med. 2017; 23:869–77. 10.1038/nm.4343 28553932 PMC5509015

[3] Siegel RL, Miller KD, Fuchs HE, Jemal A. Cancer statistics, 2022. CA Cancer J Clin. 2022; 72:7–33. 10.3322/caac.21708 35020204

[4] Wan X, Zhang Y, Tan C, Zeng X, Peng L. First-line Nivolumab Plus Ipilimumab vs Sunitinib for Metastatic Renal Cell Carcinoma: A Cost-effectiveness Analysis. JAMA Oncol. 2019; 5:491–6. 10.1001/jamaoncol.2018.7086 30789633 PMC6459127

[5] Zall H, Weber A, Besch R, Zantl N, Häcker G. Chemotherapeutic drugs sensitize human renal cell carcinoma cells to ABT-737 by a mechanism involving the Noxa-dependent inactivation of Mcl-1 or A1. Mol Cancer. 2010; 9:164. 10.1186/1476-4598-9-164 20576107 PMC2901261

[6] Capitanio U, Montorsi F. Renal cancer. Lancet. 2016; 387:894–906. 10.1016/S0140-6736(15)00046-X 26318520

[7] Choueiri TK, Motzer RJ. Systemic Therapy for Metastatic Renal-Cell Carcinoma. N Engl J Med. 2017; 376:354–66. 10.1056/NEJMra1601333 28121507

[8] Sun Z, Li T, Xiao C, Zou S, Zhang M, Zhang Q, Wang Z, Zhan H, Wang H. Prediction of overall survival based upon a new ferroptosis-related gene signature in patients with clear cell renal cell carcinoma. World J Surg Oncol. 2022; 20:120. 10.1186/s12957-022-02555-9 35422048 PMC9008912

[9] Barthel FP, Wei W, Tang M, Martinez-Ledesma E, Hu X, Amin SB, Akdemir KC, Seth S, Song X, Wang Q, Lichtenberg T, Hu J, Zhang J, et al. Systematic analysis of telomere length and somatic alterations in 31 cancer types. Nat Genet. 2017; 49:349–57. 10.1038/ng.3781 28135248 PMC5571729

[10] Lin CG, Näger AC, Lunardi T, Vančevska A, Lossaint G, Lingner J. The human telomeric proteome during telomere replication. Nucleic Acids Res. 2021; 49:12119–35. 10.1093/nar/gkab1015 34747482 PMC8643687

[11] Armanios M. The Role of Telomeres in Human Disease. Annu Rev Genomics Hum Genet. 2022; 23:363–81. 10.1146/annurev-genom-010422-091101 35609925 PMC10111244

[12] Cheng F, Luk AO, Shi M, Huang C, Jiang G, Yang A, Wu H, Lim CKP, Tam CHT, Fan B, Lau ESH, Ng ACW, Wong KK, et al. Shortened Leukocyte Telomere Length Is Associated With Glycemic Progression in Type 2 Diabetes: A Prospective and Mendelian Randomization Analysis. Diabetes Care. 2022; 45:701–9. 10.2337/dc21-1609 35085380 PMC8918237

[13] Schroder JD, de Araújo JB, de Oliveira T, de Moura AB, Fries GR, Quevedo J, Réus GZ, Ignácio ZM. Telomeres: the role of shortening and senescence in major depressive disorder and its therapeutic implications. Rev Neurosci. 2021; 33:227–55. 10.1515/revneuro-2021-0070 34388328

[14] Maciejowski J, de Lange T. Telomeres in cancer: tumour suppression and genome instability. Nat Rev Mol Cell Biol. 2017; 18:175–86. 10.1038/nrm.2016.171 28096526 PMC5589191

[15] Li SC, Jia ZK, Yang JJ, Ning XH. Telomere-related gene risk model for prognosis and drug treatment efficiency prediction in kidney cancer. Front Immunol. 2022; 13:975057. 10.3389/fimmu.2022.975057 36189312 PMC9523360

[16] Morais M, Dias F, Teixeira AL, Medeiros R. Telomere Length in Renal Cell Carcinoma: The Jekyll and Hyde Biomarker of Ageing of the Kidney. Cancer Manag Res. 2020; 12:1669–79. 10.2147/CMAR.S211225 32184670 PMC7064280

[17] Zhang Y, Huang YX, Wang DL, Yang B, Yan HY, Lin LH, Li Y, Chen J, Xie LM, Huang YS, Liao JY, Hu KS, He JH, et al. LncRNA DSCAM-AS1 interacts with YBX1 to promote cancer progression by forming a positive feedback loop that activates FOXA1 transcription network. Theranostics. 2020; 10:10823–37. 10.7150/thno.47830 32929382 PMC7482804

[18] Arraiano CM. Regulatory noncoding RNAs: functions and applications in health and disease. FEBS J. 2021; 288:6308–9. 10.1111/febs.16027 34153158

[19] Fedorko M, Febu, Bohušová J, Poprach A, Pacík D. Long non-coding RNAs and renal cell carcinoma. Klin Onkol. 2020; 33:340–9. 10.14735/amko2020340 33108878

[20] Ljungberg B, Campbell SC, Choi HY, Jacqmin D, Lee JE, Weikert S, Kiemeney LA. The epidemiology of renal cell carcinoma. Eur Urol. 2011; 60:615–21. 10.1016/j.eururo.2011.06.049 21741761

[21] Martens-Uzunova ES, Böttcher R, Croce CM, Jenster G, Visakorpi T, Calin GA. Long noncoding RNA in prostate, bladder, and kidney cancer. Eur Urol. 2014; 65:1140–51. 10.1016/j.eururo.2013.12.003 24373479

[22] Popławski P, Bogusławska J, Hanusek K, Piekiełko-Witkowska A. Nucleolar Proteins and Non-Coding RNAs: Roles in Renal Cancer. Int J Mol Sci. 2021; 22:13126. 10.3390/ijms222313126 34884928 PMC8658237

[23] Zhou X, Yao L, Zhou X, Cong R, Luan J, Wei X, Zhang X, Song N. Pyroptosis-Related lncRNA Prognostic Model for Renal Cancer Contributes to Immunodiagnosis and Immunotherapy. Front Oncol. 2022; 12:837155. 10.3389/fonc.2022.837155 35860590 PMC9291251

[24] Xu S, Liu D, Chang T, Wen X, Ma S, Sun G, Wang L, Chen S, Xu Y, Zhang H. Cuproptosis-Associated lncRNA Establishes New Prognostic Profile and Predicts Immunotherapy Response in Clear Cell Renal Cell Carcinoma. Front Genet. 2022; 13:938259. 10.3389/fgene.2022.938259 35910212 PMC9334800

[25] Zhang YP, Cheng YB, Li S, Zhao N, Zhu ZH. An epithelial-mesenchymal transition-related long non-coding RNA signature to predict overall survival and immune microenvironment in kidney renal clear cell carcinoma. Bioengineered. 2021; 12:555–64. 10.1080/21655979.2021.1880718 33517850 PMC8806254

[26] Wang L, Wang H, Wu B, Zhang C, Yu H, Li X, Wang Q, Shi X, Fan C, Wang D, Luo J, Yang J. Long Noncoding RNA *LINC00551* Suppresses Glycolysis and Tumor Progression by Regulating *c*-*Myc*-Mediated *PKM2* Expression in Lung Adenocarcinoma. Onco Targets Ther. 2020; 13:11459–70. 10.2147/OTT.S273797 33204101 PMC7665500

[27] Li C, Li Z, Yi H, Liu Z. IncRNA Linc00511 Upregulation Elevates TGFBR1 and Participates in the Postoperative Distant Recurrence of Non-Small-Cell Lung Cancer by Targeting miR-98-5p. Crit Rev Eukaryot Gene Expr. 2022; 32:1–10. 10.1615/CritRevEukaryotGeneExpr.2022039498 35993940

[28] Wu J, Cai Y, Zhao G, Li M. A ten N6-methyladenosine-related long non-coding RNAs signature predicts prognosis of triple-negative breast cancer. J Clin Lab Anal. 2021; 35:e23779. 10.1002/jcla.23779 33934391 PMC8183938

[29] Sonkar K, Ayyappan V, Tressler CM, Adelaja O, Cai R, Cheng M, Glunde K. Focus on the glycerophosphocholine pathway in choline phospholipid metabolism of cancer. NMR Biomed. 2019; 32:e4112. 10.1002/nbm.4112 31184789 PMC6803034

[30] Morais C, Gobe G, Johnson DW, Healy H. The emerging role of nuclear factor kappa B in renal cell carcinoma. Int J Biochem Cell Biol. 2011; 43:1537–49. 10.1016/j.biocel.2011.08.003 21854869

[31] Qi Y, Xia Y, Lin Z, Qu Y, Qi Y, Chen Y, Zhou Q, Zeng H, Wang J, Chang Y, Bai Q, Wang Y, Zhu Y, et al. Tumor-infiltrating CD39^+^CD8^+^ T cells determine poor prognosis and immune evasion in clear cell renal cell carcinoma patients. Cancer Immunol Immunother. 2020; 69:1565–76. 10.1007/s00262-020-02563-2 32306075 PMC11027701

[32] Jiang P, Gu S, Pan D, Fu J, Sahu A, Hu X, Li Z, Traugh N, Bu X, Li B, Liu J, Freeman GJ, Brown MA, et al. Signatures of T cell dysfunction and exclusion predict cancer immunotherapy response. Nat Med. 2018; 24:1550–8. 10.1038/s41591-018-0136-1 30127393 PMC6487502

[33] Cheng Y, Wang X, Qi P, Liu C, Wang S, Wan Q, Liu Y, Su Y, Jin L, Liu Y, Li C, Sang X, Yang L, et al. Tumor Microenvironmental Competitive Endogenous RNA Network and Immune Cells Act as Robust Prognostic Predictor of Acute Myeloid Leukemia. Front Oncol. 2021; 11:584884. 10.3389/fonc.2021.584884 33898304 PMC8063692

[34] Samstein RM, Lee CH, Shoushtari AN, Hellmann MD, Shen R, Janjigian YY, Barron DA, Zehir A, Jordan EJ, Omuro A, Kaley TJ, Kendall SM, Motzer RJ, et al. Tumor mutational load predicts survival after immunotherapy across multiple cancer types. Nat Genet. 2019; 51:202–6. 10.1038/s41588-018-0312-8 30643254 PMC6365097

[35] Zhang Q, Huang Y, Xia Y, Liu Y, Gan J. Cuproptosis-related lncRNAs predict the prognosis and immune response in hepatocellular carcinoma. Clin Exp Med. 2022. [Epub ahead of print]. 10.1007/s10238-022-00892-3 36153416

[36] Borchiellini D, Maillet D. Clinical activity of immunotherapy-based combination first-line therapies for metastatic renal cell carcinoma: the right treatment for the right patient. Bull Cancer. 2022; 109:2S4–18. 10.1016/S0007-4551(22)00234-X 35760470

[37] Hammers HJ, Plimack ER, Infante JR, Rini BI, McDermott DF, Lewis LD, Voss MH, Sharma P, Pal SK, Razak ARA, Kollmannsberger C, Heng DYC, Spratlin J, et al. Safety and Efficacy of Nivolumab in Combination With Ipilimumab in Metastatic Renal Cell Carcinoma: The CheckMate 016 Study. J Clin Oncol. 2017; 35:3851–8. 10.1200/JCO.2016.72.1985 28678668 PMC7587408

[38] Braun DM, Chung I, Kepper N, Deeg KI, Rippe K. TelNet - a database for human and yeast genes involved in telomere maintenance. BMC Genet. 2018; 19:32. 10.1186/s12863-018-0617-8 29776332 PMC5960154

[39] Ritchie ME, Phipson B, Wu D, Hu Y, Law CW, Shi W, Smyth GK. limma powers differential expression analyses for RNA-sequencing and microarray studies. Nucleic Acids Res. 2015; 43:e47. 10.1093/nar/gkv007 25605792 PMC4402510

[40] Subramanian A, Tamayo P, Mootha VK, Mukherjee S, Ebert BL, Gillette MA, Paulovich A, Pomeroy SL, Golub TR, Lander ES, Mesirov JP. Gene set enrichment analysis: a knowledge-based approach for interpreting genome-wide expression profiles. Proc Natl Acad Sci U S A. 2005; 102:15545–50. 10.1073/pnas.0506580102 16199517 PMC1239896

[41] Yoshihara K, Shahmoradgoli M, Martínez E, Vegesna R, Kim H, Torres-Garcia W, Treviño V, Shen H, Laird PW, Levine DA, Carter SL, Getz G, Stemke-Hale K, et al. Inferring tumour purity and stromal and immune cell admixture from expression data. Nat Commun. 2013; 4:2612. 10.1038/ncomms3612 24113773 PMC3826632

[42] Mayakonda A, Lin DC, Assenov Y, Plass C, Koeffler HP. Maftools: efficient and comprehensive analysis of somatic variants in cancer. Genome Res. 2018; 28:1747–56. 10.1101/gr.239244.118 30341162 PMC6211645

[43] Charoentong P, Finotello F, Angelova M, Mayer C, Efremova M, Rieder D, Hackl H, Trajanoski Z. Pan-cancer Immunogenomic Analyses Reveal Genotype-Immunophenotype Relationships and Predictors of Response to Checkpoint Blockade. Cell Rep. 2017; 18:248–62. 10.1016/j.celrep.2016.12.019 28052254

[44] Maeser D, Gruener RF, Huang RS. oncoPredict: an R package for predicting in vivo or cancer patient drug response and biomarkers from cell line screening data. Brief Bioinform. 2021; 22:bbab260. 10.1093/bib/bbab260 34260682 PMC8574972

