# Supplementary material for: A comprehensive prognostic and immunological analysis of telomere-related lncRNAs in kidney renal clear cell carcinoma
Source: Aging (Albany NY). 2023 Oct 16;15(20):11012–32. doi: 10.18632/aging.205056 (PMC10637817; doi:10.18632/aging.205056)
Supplement: Supplementary Figures [file aging-15-205056-s001.pdf]

SUPPLEMENTARY FIGURES

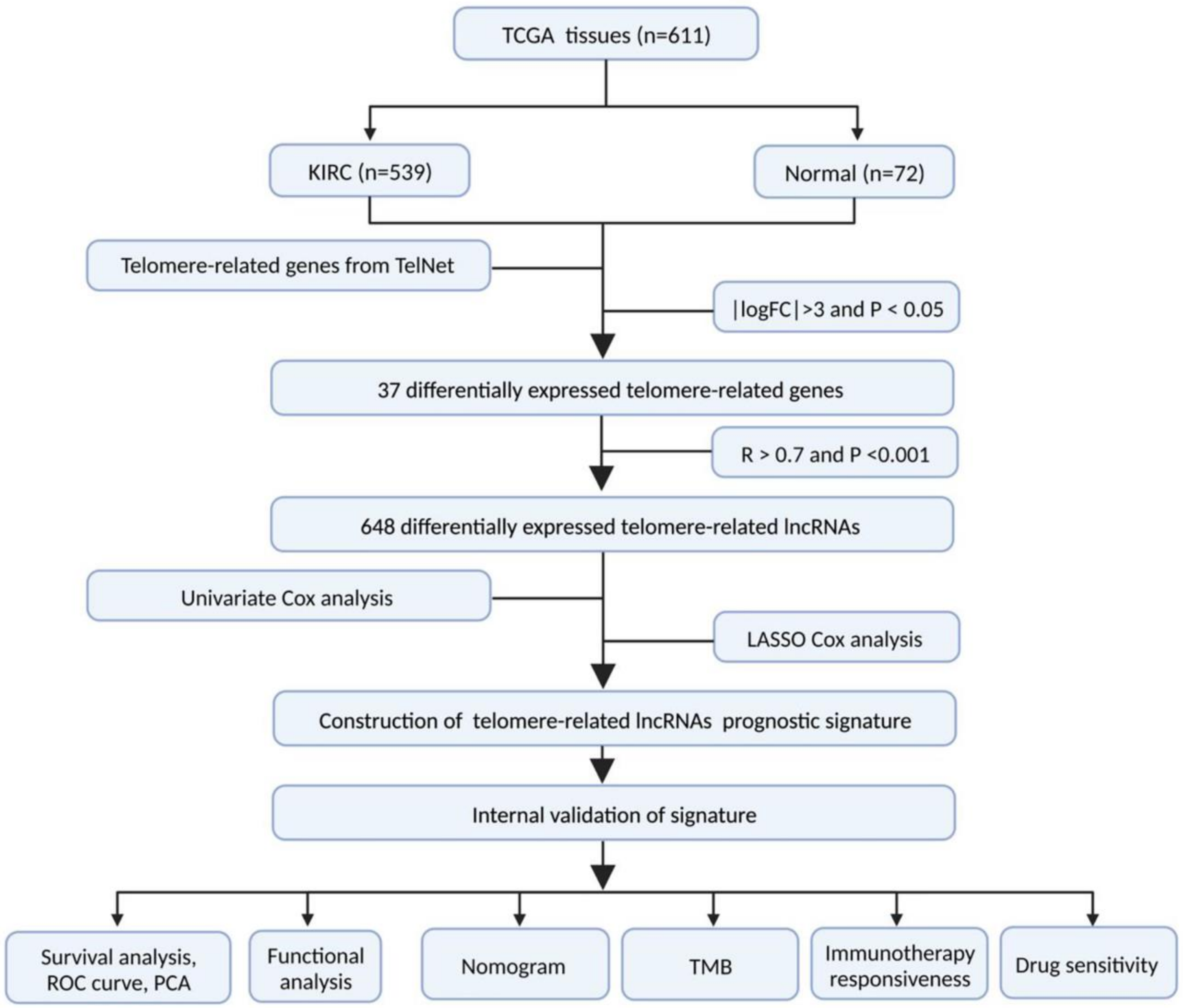

Supplementary Figure 1. The flowchart of our research.

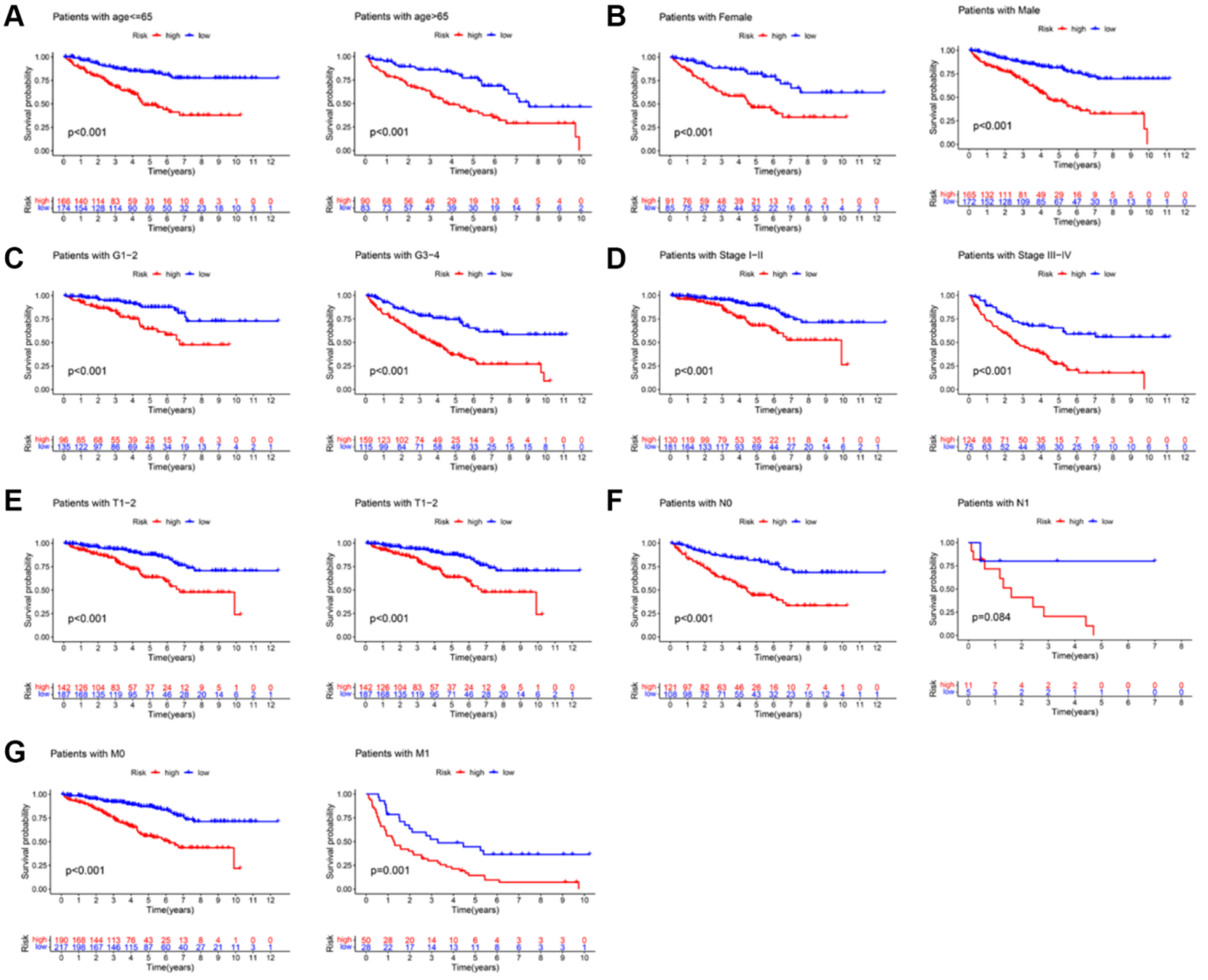

Supplementary Figure 2. Stratified survival analysis based on TRLs prognostic signature. The OS rates of different risk groups with different clinical features; including age (**A**); gender (**B**); grade (**C**); AJCC stage (**D**); T stage (**E**); N stage (**F**); and M stage (**G**).

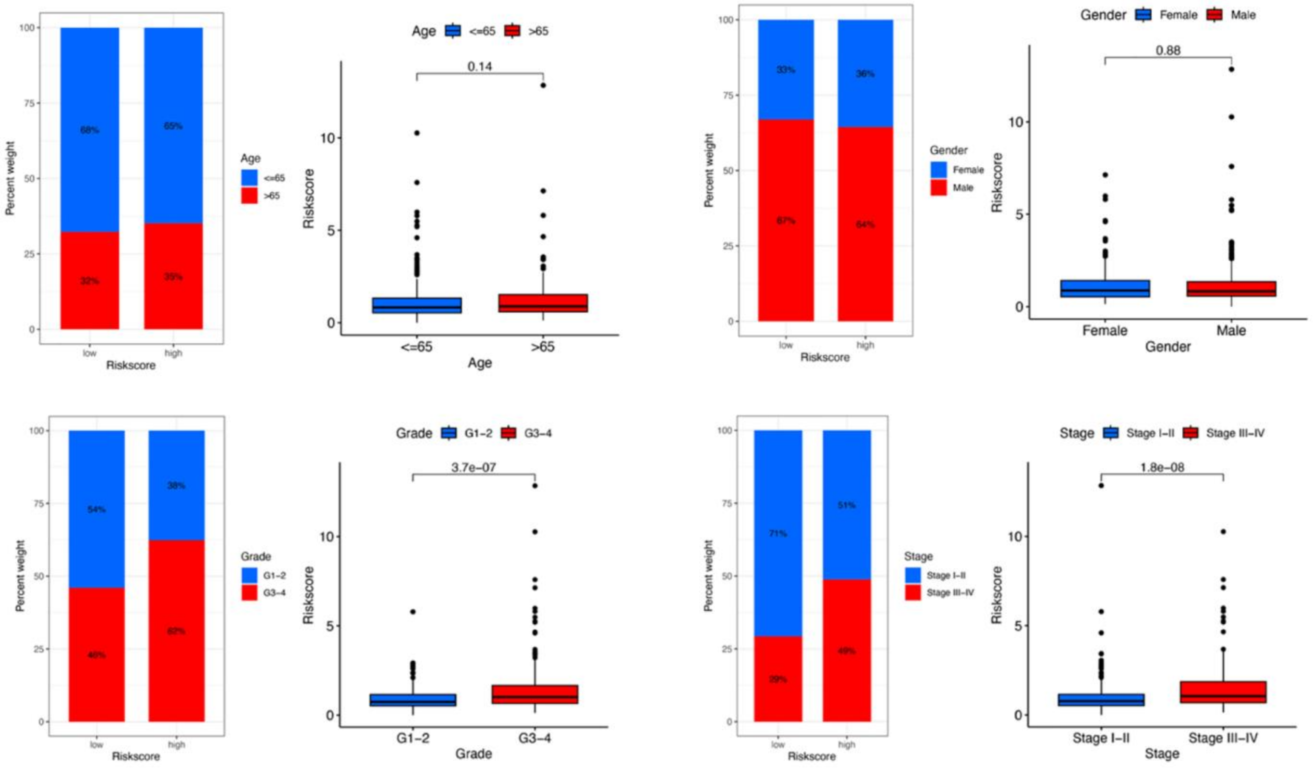

Supplementary Figure 3. The distribution of the risk scores in terms of age, gender, grade, and stage.

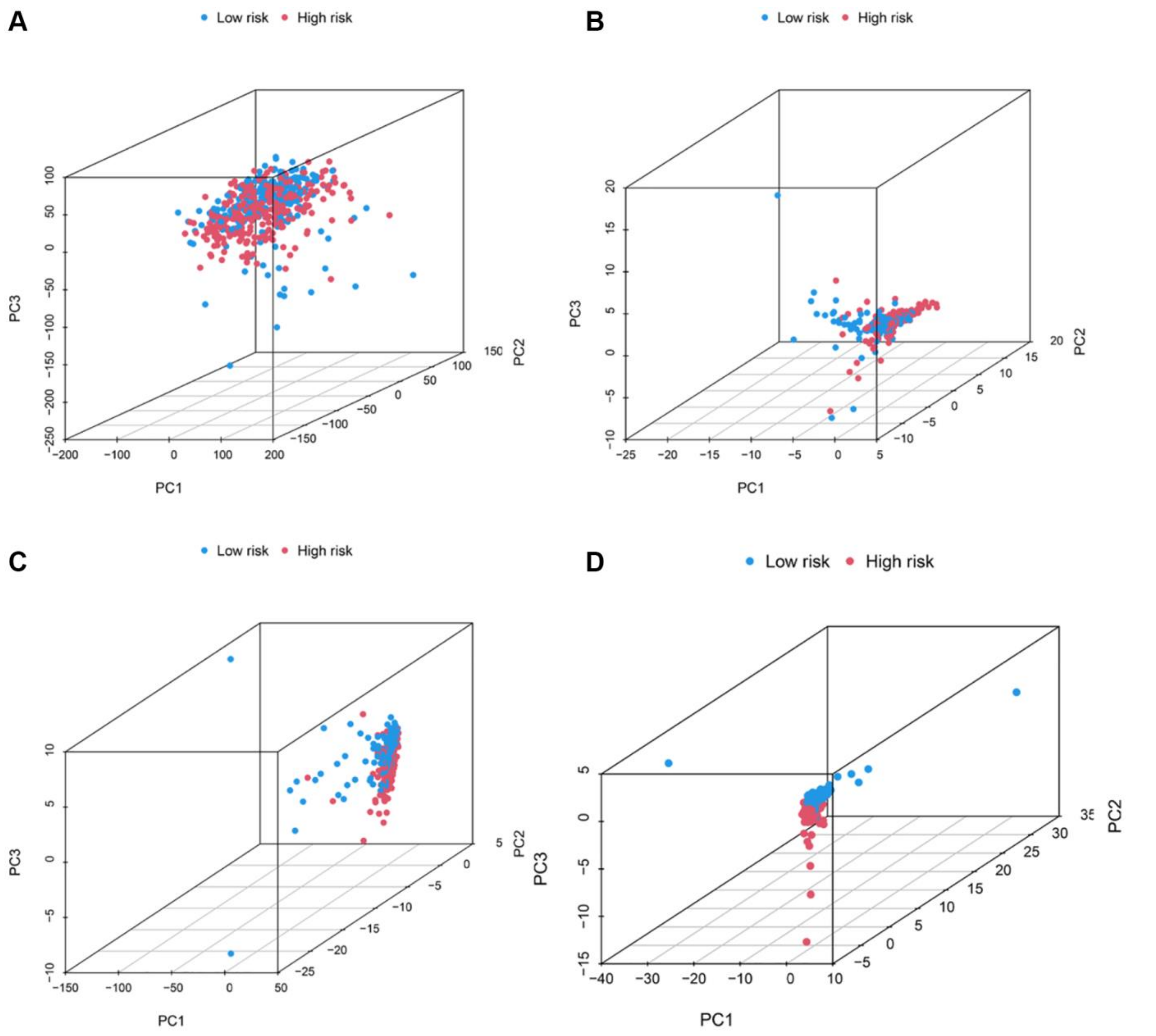

Supplementary Figure 4. Principal components analysis between low- and high-risk groups with different data sets. (**A**) The whole genome data. (**B**) The differentially expressed telomere-related genes. (**C**) TRLs. (**D**) The signature-based TRLs.

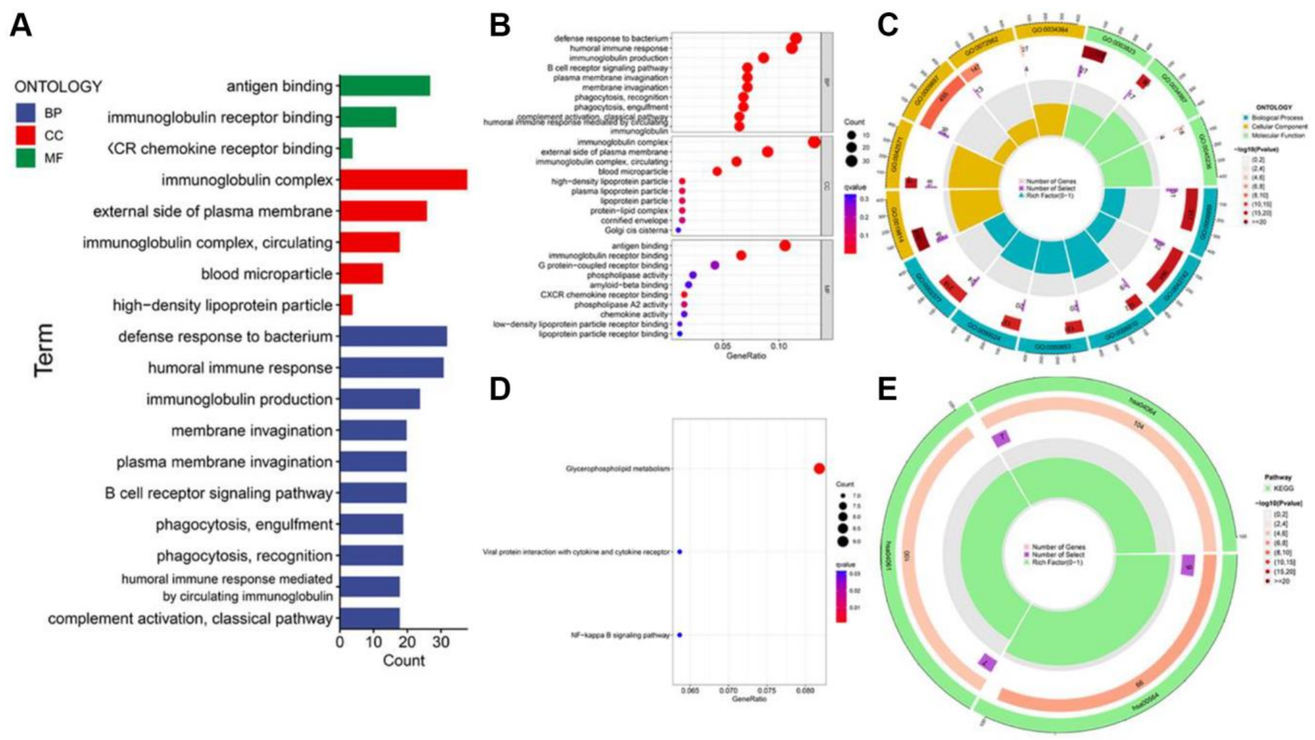

Supplementary Figure 5. Functional enrichment analysis of 548 genes with differential expression between low- and high-risk groups. (**A**–**C**) GO enrichment analysis. (**D**, **E**) KEGG enrichment analysis.
