# Supplementary material for: A comprehensive prognostic and immunological analysis of telomere-related lncRNAs in kidney renal clear cell carcinoma
Source: Aging (Albany NY). 2023 Oct 16;15(20):11012–32. doi: 10.18632/aging.205056 (PMC10637817; doi:10.18632/aging.205056)
Supplement: Supplementary Table 1 [file aging-15-205056-s002.pdf]

SUPPLEMENTARY TABLE

Supplementary Table 1. Characteristics of all patients included in this study.

Variable	Entire cohort (*n* = 513)	Training cohort (*n* = 257)	Testing cohort (*n* = 256)
	Number (%)	Number (%)	Number (%)
**Age**			
≤65	340 (66.28%)	168 (65.62%)	172 (66.93%)
>65	173 (33.72%)	88 (34.38%)	85 (33.07%)
**Gender**			
Female	176 (34.31%)	91 (35.55%)	85 (33.07%)
Male	337 (65.69%)	165 (64.45%)	172 (66.93%)
**Grade**			
G1	12 (2.34%)	11 (4.3%)	1 (0.39%)
G2	219 (42.69%)	109 (42.58%)	110 (42.8%)
G3	201 (39.18%)	94 (36.72%)	107 (41.63%)
G4	73 (14.23%)	38 (14.84%)	35 (13.62%)
Gx	5 (0.97%)	2 (0.78%)	3 (1.17%)
Unknow	3 (0.58%)	2 (0.78%)	1 (0.39%)
**AJCC stage**			
I	255 (49.71%)	127 (49.61%)	128 (49.81%)
II	56 (10.92%)	29 (11.33%)	27 (10.51%)
III	117 (22.81%)	50 (19.53%)	67 (26.07%)
IV	82 (15.98%)	49 (19.14%)	33 (12.84%)
Unknow	3 (0.58%)	1 (0.39%)	2 (0.78%)
**T stage**			
T1	261 (50.88%)	133 (51.95%)	128 (49.81%)
T2	68 (13.26%)	38 (14.84%)	30 (11.67%)
T3	173 (33.72%)	79 (30.86%)	94 (36.58%)
T4	11 (2.14%)	6 (2.34%)	5 (1.95%)
**N stage**			
N0	229 (44.64%)	104 (40.62%)	125 (48.64%)
N1	16 (3.12%)	11 (4.3%)	5 (1.95%)
Nx	268 (52.24%)	141 (55.08%)	127 (49.42%)
**M stage**			
M0	407 (79.34%)	194 (75.78%)	213 (82.88%)
M1	78 (15.2%)	45 (17.58%)	33 (12.84%)
Mx	26 (5.07%)	16 (6.25%)	10 (3.89%)
Unknow	2 (0.39%)	1 (0.39%)	1 (0.39%)
